# Humoral and cellular immunity to SARS-CoV-2 Ancestral and Omicron BA.5 variants following vaccination in myelofibrosis patients

**DOI:** 10.1038/s41408-023-00824-8

**Published:** 2023-04-10

**Authors:** Ahmad Alcheikh, Griffith B. Perkins, Phillippa A. Pucar, Amelia Cecchin, Cheng Sheng Chai, Matthew Tunbridge, Anouschka Akerman, Anupriya Aggarwal, Vanessa Milogiannakis, Stuart Turville, Sharon Allen, Pravin Hissaria, Tatjana Banovic, P. Toby Coates, David M. Ross

**Affiliations:** 1 Department of Haematology, Royal Adelaide Hospital, Adelaide, Australia; 2 Central and Northern Adelaide Renal and Transplantation Service, Royal Adelaide Hospital, Adelaide, Australia; 3 Immunology Directorate, SA Pathology, Adelaide, Australia; 4 Adelaide Medical School, University of Adelaide, Adelaide, Australia; 5 Department of Immunology, Royal Adelaide Hospital, Adelaide, Australia; 6 Kirby Institute, University of New South Wales, Sydney, Australia; 7 Haematology Directorate, SA Pathology, Adelaide, Australia; 8 Department of Haematology, Flinders University and Medical Centre, Adelaide, Australia

**Keywords:** Myeloproliferative disease, Infectious diseases, Adaptive immunity, Preventive medicine, T cells

**Dear Editor,**

Myelofibrosis (MF) is a clonal myeloproliferative neoplasm associated with inflammatory manifestations including fibrosis and constitutional symptoms. The standard treatment for symptomatic MF is ruxolitinib, a JAK1/2 inhibitor (JAKi) that antagonizes cytokine receptor signalling. JAK-dependent cytokine signals are integral to an effective inflammatory response and ruxolitinib treatment is accompanied by an increased risk of infection, including reactivation of varicella zoster virus and tuberculosis [1]. Individuals with advanced MF have an increased risk of severe COVID-19, and impaired response to vaccination [2–6].

In response to the COVID-19 pandemic, Australia implemented strict isolation measures. Negligible community transmission of SARS-CoV-2 until November 2021 in South Australia provided the opportunity to assess vaccine responses with minimal interference from natural infection.

Adult patients with primary or secondary MF receiving a JAKi were recruited for a longitudinal observational study of vaccine response. The study was approved by the relevant ethics committee and clinical data extracted from health records. Patients with MF were prioritized for early vaccination, and most participants received two initial doses of the viral vector-based AZD1222 (University of Oxford/AstraZeneca), followed by a third dose of an mRNA-platform vaccine, BNT162b2 (Pfizer/BioNTech) or mRNA-1273 (Moderna/NAIAD) [7–9]. Participants provided samples at timepoints before and after vaccine doses (Fig. 1A). Cellular and humoral immune responses to the original two-dose vaccine schedule were compared to those of 10 healthy controls (HC) of comparable age and sex (Supplementary Table 1). Upon recommendation of a third vaccine dose, the MF cohort was expanded to include individuals receiving alternative therapies other than JAKi, and additional samples were requested within the 14 days preceding (T3), and 28 days following (T4), the third dose. Serological immunity was assessed by SARS-CoV-2 Spike-specific IgG ELISA and live virus neutralization of Ancestral and Omicron BA.5 variants, and T cell immunity by IFNγ-ELISpot.

**Fig. 1:**
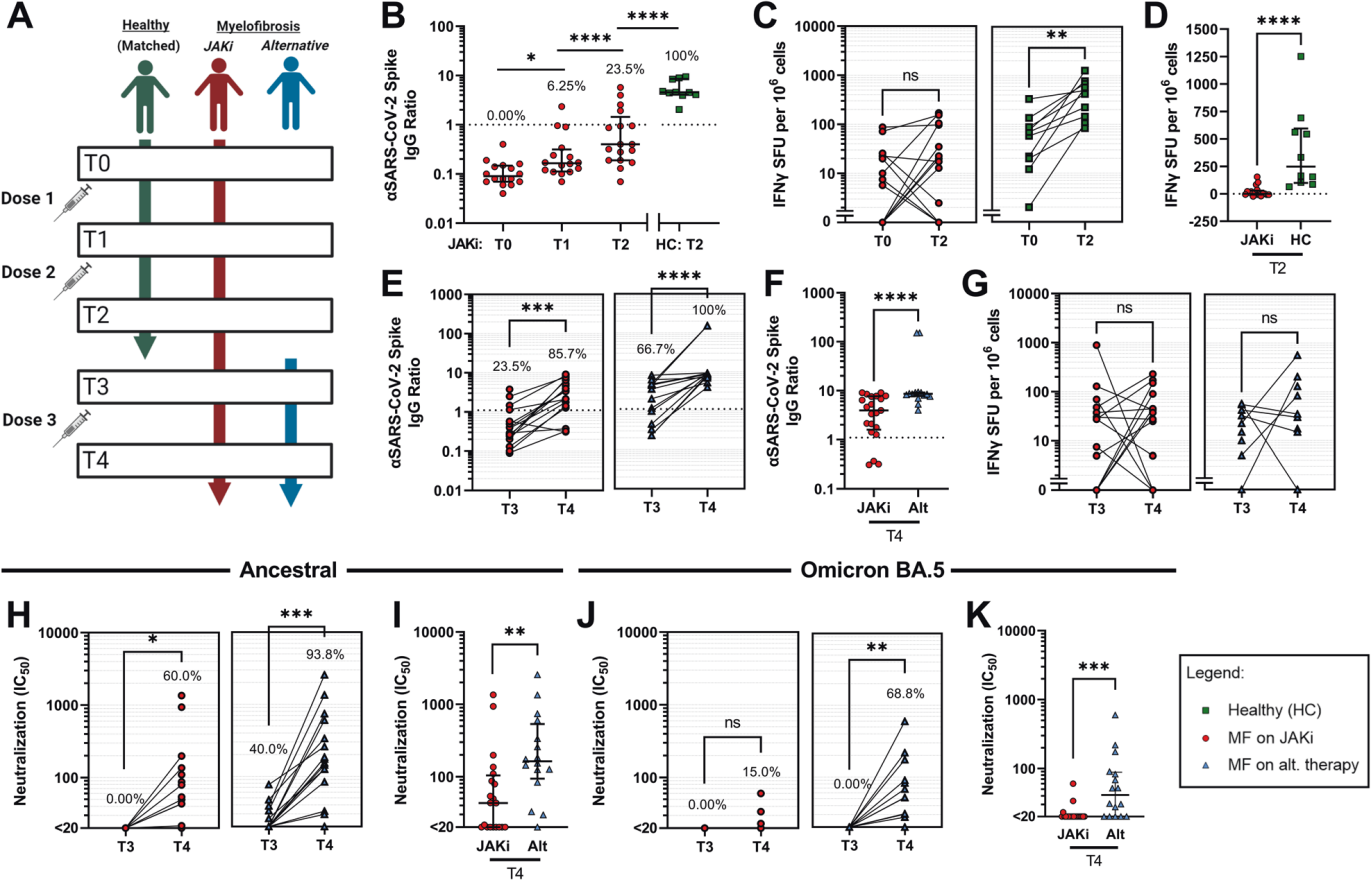
JAK inhibitor use impairs humoral and cellular immunity to COVID-19 vaccination and limits third-dose protective immunity against Ancestral and Omicron BA.5 variants in myelofibrosis. **A** Study design. Immunogenicity of the standard two-dose vaccine schedule was assessed in individuals with myelofibrosis receiving a JAK inhibitor compared against healthy controls of comparable age, sex distribution and vaccine selection. The effect of a third vaccine dose on correlates of protective immunity against Ancestral and Omicron BA.5 variants was assessed for myelofibrosis patients receiving a JAK inhibitor compared with those receiving alternative (Alt) therapies. **B** Anti-SARS-CoV-2 Spike IgG titers (EUROIMMUN) in myelofibrosis patients receiving a JAK inhibitor, assessed at baseline, and pre- (T1) and post- (T2) administration of a second vaccine dose. Titers measured after a second dose (median 15 days; IQR 14–20.5) were compared with those of healthy controls (median 21 days; IQR 20–21.5). *P* values from paired comparisons, from left to right, were 0.017 (Wilcoxon test), <0.0001 (Wilcoxon test) and <0.0001 (Mann–Whitney U test). **C** Assessment of functional SARS-CoV-2 Spike-reactive T cells by IFNγ ELISpot. Peripheral blood mononuclear cells collected from MF patients on JAK inhibitors and healthy controls at baseline and after 2 vaccine doses were stimulated for 18 h with overlapping peptides spanning the full length of the SARS-CoV-2 Spike protein, and cellular immunity assessed as IFNγ spot-forming units by ELISpot. **D** Cellular immune response to a two-dose vaccination schedule in myelofibrosis patients receiving a JAKi and healthy individuals. Change in spot counts from baseline to T2 were calculated in order to account for pre-existing immunity measured in some individuals prior to vaccination. **E** Anti-SARS-CoV-2 Spike IgG titers (EUROIMMUN) in myelofibrosis patients receiving a JAK inhibitor or alternative therapy pre- and post-administration of a third vaccine dose. **F** Comparison of anti-SARS-CoV-2 Spike IgG titers (EUROIMMUN) in myelofibrosis patients receiving a JAK inhibitor or alternative therapy following a third vaccine dose. **G** Functional SARS-CoV-2 Spike-reactive T cell counts in myelofibrosis patients receiving a JAK inhibitor or alternative therapy pre- and post-administration of a third vaccine dose. **H** Serological neutralization of live SARS-CoV-2 virus Ancestral variant. Percentage of individuals achieving effective neutralization (IC50 ≥ 20) is indicated. **I** Capacity for neutralization of the Ancestral variant following a third vaccine dose in myelofibrosis patients compared by treatment. **J** Serological neutralization of live SARS-CoV-2 virus Omicron BA.5 variant. Percentage of individuals achieving effective neutralization (IC50 ≥ 20) is indicated. **K** Capacity for neutralization of the Omicron BA.5 variant following a third vaccine dose in myelofibrosis patients compared by treatment. Significant difference between time points by Wilcoxon signed-rank test or Student’s Paired T test, and between groups by Mann–Whitney U test: ns, non-significant; *p < 0.05; **p < 0.01; ***p < 0.001; ****p < 0.0001. For values, see Supplementary Table 3.

Forty patients contributed samples with median follow-up of 356 days after the first vaccine. Patient characteristics are shown in Supplementary Table 2. Patients on a JAKi had features of advanced disease compared to those on alternative therapies, with higher clinical scores (by Dynamic International Prognostic Scoring System-Plus; DIPSS+), lower haemoglobin and platelet counts, and higher LDH. Twenty-four patients were on a JAKi: ruxolitinib (*n* = 21, median dose 10 mg bd); momelotinib 200 mg/d (*n* = 2); or fedratinib 400 mg/d (*n* = 1). Sixteen patients were on hydroxyurea (*n* = 8) or no cytoreductive therapy (*n* = 8). Three patients were in remission following allogeneic stem cell transplantation (3, 4 and 8 years prior), one of whom was on ruxolitinib and ciclosporin for chronic graft-vs-host disease (included in the JAKi cohort). Four patients commenced a JAKi after commencing vaccination, one after the first dose and three after the second dose and were included in the non-JAKi cohort.

MF patients receiving a JAKi demonstrated severely impaired humoral and cellular immune responses to the initial two-dose vaccination schedule relative to healthy individuals. Seroconversion (EUROIMMUN ratio ≥1.1) occurred after the first vaccine dose (T1) in 1/16 patients (6.3%) on JAKi, rising to 4/17 (23.5%) after the second dose (Fig. 1B; Supplementary Table 3). By comparison all HC seroconverted following two doses of AZD1222 (median ratio 4.56 vs 0.40 in MF patients on JAKi, *p* < 0.0001) (Fig. 1B). Frequency of Spike-reactive IFNγ-secreting T cells was also significantly reduced compared with HC following two doses (median 18.75 [IQR 0–103.2] vs 458 [IQR 134.5–702] SFU per 10^6^ cells; *p* < 0.0001), as was the change from baseline, a more accurate measure of the magnitude of vaccine response (median 0 vs 248.0, respectively; *p* < 0.0001) (Fig. 1C, D).

Given the severely impaired immunogenicity of a two-dose vaccination schedule in MF patients receiving a JAKi, we evaluated the response of this group to a third (mRNA-platform) dose relative to a cohort of MF patients receiving alternative therapies. Mean interval from the first dose to T3 and T4 was similar between JAKi and non-JAKi (190 vs 187 days, *p* = 0.30, and 225 vs 229 days, *p* = 0.81, respectively). Prior to a third dose, fewer patients on JAKi therapy seroconverted than in MF patients on alternative therapies (24% vs 67%; *p* = 0.031) (Supplementary Fig. 1A–F). A third dose significantly increased median anti-Spike IgG titers in both groups (Fig. 1E), but titers remained lower in those receiving a JAKi (3.96 vs 8.61; *p* < 0.0001) (Fig. 1F). T cell responses did not improve with a third dose in either group (Fig. 1G).

A significant limitation of COVID-19 vaccine research has been interpreting real-world protection from immunogenicity data. Early in the pandemic, Khoury and colleagues [10] described the close correlation between serological neutralization of live SARS-CoV-2 virus and real-world protection from infection. In this data set, 50% protection from infection was achieved at a neutralization titer (IC_50_) equivalent to 20.2% of the mean titer of a cohort of healthy convalescent individuals (infected with the Wuhan strain). In order to estimate the level of protection afforded patients in the present study, we measured serological neutralization for 20 healthy convalescent donors from the first SARS-CoV-2 wave in Australia, and defined an end-point titer of ≥20 as an effective neutralization threshold for protective immunity (see ‘Methods’).

Following a third vaccine dose, effective neutralization of Ancestral SARS-CoV-2 (A.2.2) increased from 0/18 to 12/20 (60%) for patients on JAKi (*p* < 0.0001) (Fig. 1H). By comparison, rates for patients on alternative therapies rose from 6/15 (40%) to 15/16 (94%; *p* = 0.0021) (Fig. 1H). Consistent with antibody escape by the Omicron BA.5 variant, neutralization of BA.5 was reduced for all groups relative to A.2.2 (Fig. 1H–K; Supplementary Fig. 2). Prior to a third dose, no MF patients on either treatment demonstrated effective neutralization of BA.5 (Fig. 1J). Following a third dose, 69% of patients on alternative therapies demonstrated effective neutralization, compared with 15% on JAKi. Patients who received at least one vaccine dose prior to commencing JAKi therapy tended to have improved serologic responses (Supplementary Fig. 3).

The emergence of SARS-CoV-2 variants that escape vaccine-induced humoral immunity has highlighted the importance of T cells [11–14]. In patients with haematological malignancies, high CD8^+^ T cell counts are associated with improved outcomes of COVID-19 despite reduced levels of virus-neutralizing antibodies [15]. T cell responses did not improve significantly after a third vaccination in MF patients. ELISpot counts correlated with anti-S IgG titer (*r* = 0.37, *p* = 0.047) and with neutralization of A.2.2 (*r* = 0.41, *p* = 0.029), suggesting that patients with a more robust serological response may have a less impaired T cell response. In a multivariate linear regression model, only JAKi therapy (*β* = −2.735, 95% CI −4.233 to −1.237; *p* = 0.0013) and total lymphocyte count (*β* = 0.9709, 95% CI 0.1609–1.781; *p* = 0.022) were found to predict T3 serologic response. Only male sex was predictive of poorer serological response at T4. JAKi treatment predicted poorer neutralization against A.2.2 at T3 and Omicron BA.5 strain at T4 (Supplementary Tables 4–10). By logistic regression and bivariate analysis, only JAKi was associated with nonresponders, with relative risk of 2.9 (95% CI 1.17–5.20, *p* = 0.031), 1.67 (1.67–1.92, *p* = 0.0045), 6.40 (1.25–37.0, *p* = 0.026) and 2.72 (1.44–6.10, *p* = 0.0017) for T3 anti-Spike IgG, T3 and T4 Ancestral neutralisation, and T4 Omicron BA.5 neutralisation, respectively (Supplementary Tables 11–19).

Ten patients (7 males, 3 females) were documented to have SARS-CoV-2 infection during the follow-up period, occurring either after the third vaccine dose (*n* = 4) or the fourth dose (*n* = 6). Of the 10 patients, 5 were on ruxolitinib (representing 21% of the JAKi cohort) and 5 were on alternative therapies (representing 31% of the non-JAKi cohort). One patient on ruxolitinib was hospitalized with moderately severe COVID-19 disease. The remainder received either no treatment or outpatient-based oral antiviral therapy, and no patients died from COVID-19.

Patients who had clinical infection had lower median T3 and T4 anti-S antibodies and neutralization of Ancestral and Omicron BA.5 virus compared to the rest of our study cohort, and only two met the threshold for effective neutralization of Omicron BA.5 after the third dose. However, the study was not powered to assess the association between humoral immunity and clinical outcomes, and these observations were not statistically significant (Supplementary Fig. 4, Supplementary Table 20).

This study describes severe impairment of humoral and cellular vaccine responses in MF, and identifies JAKi use as a modifiable predictor of inadequate protection against SARS-CoV-2 strains. Following a third vaccine dose, 85% of patients on a JAKi (and >30% on alternative therapies) did not demonstrate effective immunity against Omicron BA.5 and T cell responses, which provide cross-protective immunity in the absence of effective neutralization, remained impaired [15]. Patients and clinicians should be aware that standard vaccination is less effective in MF so that simple hygiene measures to reduce the risk of exposure to SARS-CoV-2 can be employed. Whenever possible, vaccination should be done prior to the commencement of JAKi therapy. We suggest patients on a JAKi be included among at-risk groups considered for access to prophylactic measures currently in development to protect against current and future viral strains.

Encouragingly, consistent increases in antibody titer were observed for the JAKi cohort with repeated vaccination, suggesting further booster dosing may help overcome this impaired response. These results should assist in the ongoing refinement of COVID-19 vaccination and management guidelines, and motivate investigation into the immunogenicity of other key vaccines in patients receiving ruxolitinib.

## Supplementary information

Supplementary Information
