# Supplementary material for: Humoral and cellular immunity to SARS-CoV-2 Ancestral and Omicron BA.5 variants following vaccination in myelofibrosis patients
Source: Blood Cancer J. 2023 Apr 10;13(1):50. doi: 10.1038/s41408-023-00824-8 (PMC10083173; doi:10.1038/s41408-023-00824-8)
Supplement: Supplementary file 1 — Supplementary Information [file 41408_2023_824_MOESM1_ESM.docx]

Humoral and cellular immunity to SARS-CoV-2 Ancestral and Omicron BA.5 variants following vaccination in myelofibrosis patients

Ahmad Alcheikh*, Griffith B Perkins*, Phillippa A Pucar, Amelia Cecchin, Cheng Sheng Chai, Matthew Tunbridge, Anouschka Akerman, Anupriya Aggarwal, Vanessa Milogiannakis, Stuart Turville, Sharon Allen, Pravin Hissaria, Tatjana Banovic, P. Toby Coates, and David M Ross

*Co-first authors

Supplementary Information

Supplementary Methods

***Study Design and Participants.*** Myelofibrosis patients who had given written informed consent to donate biological samples to the South Australian Cancer Research Biobank (SACRB) were invited to provide additional blood samples before and after vaccination. Adult patients with a diagnosis of primary or post-polycythaemia vera (PPV)/post-essential thrombocythaemia (PET) MF who had at least one dose of any COVID-19 vaccine were eligible for inclusion if they provided at least one sample after vaccination. The study was approved by the Central Adelaide Local Health Network (CALHN) Human Research Ethics Committee and clinical data were extracted from health records.

Patients were invited to provide samples within 30 days before the first vaccine, within 14 days before the second vaccine, and approximately 14 days after the second vaccine. The invitation was initially sent to patients on JAK inhibitors and subsequently extended to all patients with MF. The majority of patients received 2 doses of AZD1222 vaccine given 12 weeks apart, while some, predominantly younger, patients received 2 doses of BNT612b2 vaccine 3 weeks apart. After the Australian government recommended a third dose of vaccine (BNT61b2 or mRNA-1273) for immunocompromised patients (recommended to be given 2-6 months after the second dose) additional samples were requested within the 14 days before and approximately 28 days after the third dose. Samples were separated into serum and EDTA tubes. Mononuclear cells were separated by centrifugation using Lymphoprep SG 1.077 g/mL, and cryopreserved in human serum albumin and DMSO.

***SARS-CoV-2 Spike-Specific IgG.*** To assess specific IgG responses to the S1 domain of the spike protein of SARS-CoV-2, a commercial enzyme-linked immunosorbent assay (ELISA) (EUROIMMUN, Lubeck, Germany) was performed on serum or plasma samples according to the manufacturer’s recommendations in the diagnostic immunology laboratory of SA Pathology. This assay has demonstrated excellent sensitivity for detecting anti-S IgG in serum following SARS-CoV-2 infection (1). The result is reported semi-quantitatively as a ratio of antibody binding compared to a calibrator sample: a ratio of <0.8 is considered negative, 0.8 to <1.1 borderline and ≥1.1 positive.

***Live Virus Neutralization.*** Live viral neutralization was carried out using the method described by Aggarwal *et al* (2) using clinical isolates and approvals previously described for Clade A.2.2 & Omicron BA.5 (2,3). HEK-ACE2/TMPRSS cells (Clone 24) (ref 4) were seeded in 384-well plates at 5 × 10^3^ cells/well in the presence of the live cell nuclear stain Hoechst-33342 dye (NucBlue, Invitrogen) at a concentration of 5% v/v. Two-fold dilutions of patient serum samples were mixed with an equal volume of SARS-CoV-2 virus solution (1.25 × 10^4^ TCID50/ml) and incubated at 37°C for 1 h before adding 40 μl, in duplicate, to the cells (final MOI = 0.05). Viral variants used were the Omicron (B.1.1.529) sub-variant BA.5, as well as the ‘wild-type’ Ancestral virus (A.2.2) from clade A and presenting no amino acid mutations in Spike (similar to Wuhan ancestral variant). Plates were incubated for 24 h post infection and entire wells were imaged by high-content fluorescence microscopy, cell counts obtained with automated image analysis software.

The threshold for effective neutralization was derived from a published value of 20.2% of the mean neutralization titre (IC_50_) of first-wave convalescent individuals (5). This value correlated with 50% protection from real-world infection early in the COVID-19 pandemic. In our study, 20 convalescent sera from an early wave were included in the same experimental run as the study samples, and 20.2% of the mean IC_50_ for this group calculated to be 14.7. As the lowest dilution factor tested for all samples was 20, effective neutralisation of both the Ancestral and Omicron BA.5 variants was defined as an IC_50_ ≥ 20.

In order to report standardized measures, the WHO reference sample Priv-874 was included in the same experimental run as the study samples. Priv-874 is 10% IgG harvested from >15k US plasma donors and is equivalent to Standard 12 (Candidate 1; 21/338) in the most recent WHO Expert Committee on Biological Standardization report for assessment of SARS-CoV-2 Ancestral neutralisation (6) Priv-874 represents a reference value of 7670 WHO IU/ml, and neutralisation titres for positive samples (IC_50_ ≥ 20) were converted to WHO units with the following formula: [Sample IC50] / [Privagen IC50 x 7670 IU/ml].

***IFNγ ELISpot.*** Functional T cell response was assessed by an in-house IFNγ ELISpot assay (7) Millipore 96-well plates with nitrocellulose membranes (Merck, Branchburg, NJ, USA) were activated with 35% ethanol for 30 s, before washing twice with PBS. Wells were coated with anti-IFNγ capture antibody (Clone 2G1, ThermoFisher, Cambridge, MA, USA) overnight at 4°C, then washed twice with PBS. PBMCs were thawed by dropwise addition of complete medium (RPMI + 20% FCS, glutamate, penicillin, streptomycin) and benzonase (Merck, Kenilworth, NJ, USA) to prevent aggregation, and rested for 2 h before counting. PBMCs were seeded at 2.5 x 10^5^ cells per well in duplicate and treated with 4 pools of overlapping peptides spanning the length of the spike protein (NR-52402, BEI Resources, Manassas, VA). PHA (5 μg/mL; Merck, Branchburg) was used as a positive control. After 18 h at 37°C, wells were washed five times with PBS, then ten times with PBS + 0.05% tween-20. Captured IFNγ was detected with a biotinylated anti-IFNγ antibody (Clone B133.5; ThermoFisher, Cambridge) at 4 °C overnight. Unbound detection antibody was removed by washing with PBS + 0.05% tween-20, and a streptavidin:HRP conjugate (BD Biosciences, NJ, USA) was added for four hours at 4 °C. AEC substrate (BD Biosciences, Franklin Lakes, NJ, USA) was added for 15 min at room temperature, before rinsing with deionized water and enumeration of spots using an ImmunoSpot analyzer and software (Cellular Technology Ltd., Bonn, Germany). Spot counts are presented as combined counts from the 4 peptide pools per 10^6^ PBMCs. All washing steps were performed using an automated plate washer. The following reagent was obtained through BEI Resources, NIAID, NIH: Peptide Array, SARS-Related Coronavirus 2 Spike (S) Glycoprotein, NR-52402.

***Statistical Analysis.*** Data analysis was performed by AA, and all authors had access to the data. All tests were two-sided. Categorical variables were compared with Chi-square or Fisher’s exact test, where appropriate. Continuous paired variables were compared using paired t test or Wilcoxon matched-pairs signed rank test, or the Mann-Whitney U and Kruskal-Wallis tests for non-Gaussian unpaired variables. Exploratory correlations were performed at an α significance level of 0.01. Other p values were assessed at a significance level of 0.05. Multiple comparisons were followed up with a Dunn’s multiple comparisons test. Correlation was performed using nonparametric Spearman correlation. Relative risks were calculated using the Koopman asymptomatic score. All analyses were conducted using GraphPad Prism 9.4.1.

***Regression Modelling.*** Multiple linear regression by least squares modelling was applied T3 and T4 result for anti-S serology, ELISpot and neutralisation data. Assumptions of normality of residuals was tested using D’Agostino-Pearson omnibus (K2) and of linearity using visual inspection of scatter plots. Variables included are age, sex (M/F), myelofibrosis type (primary (PMF) or secondary (SMF)), DIPPS-plus score, spleen size around time of first vaccine (cm), JAKi therapy (Yes/No), absolute lymphocyte count (x10^9^/L). Results of the linear regression modelling for each outcome variable are shown in Supplementary Tables 4-10 with accompanying scatterplots. The main limitation relates to linearity of the variables, which appeared to be imperfect (see scatterplots), and for T3 ELISpot and T3 neutralisation of the Ancestral strain data, residuals did not follow normal distribution (D'Agostino-Pearson omnibus K2 test). A model could not be fitted for T3 Omicron BA.5 neutralisation as no patient showed neutralisation activity. For patients on JAKi, linear regression modelling was attempted incorporating dose of JAKi but none of these models were predictive (data not shown). Likewise for patients who had lymphocyte subsets measured, useful models could not be constructed, due to too few data points (data not shown). Multiple logistic regression modelling for bivariate outcomes did not converge; simple logistic regression was performed instead. All analyses were conducted using GraphPad Prism 9.4.1.

Supplementary Tables (and regression plots)

**Supplementary Table 1. Characteristics of healthy controls and MF patients on JAKi at T2.**

|  | **Control** | **MF patients** | **p-value** | **Test** |
| --- | --- | --- | --- | --- |
| Number (n) | 10 | 17 |  |  |
| Age (Mean ± SD) | 69.07 ± 4.980 | 71.29 ± 7.312 | 0.40 | Unpaired t test |
| Sex (Male) | 5/10 (50%) | 14/17 (82.4%) | 0.10 | Fisher’s exact test |

**Supplementary Table 2: Patient characteristics at time of first SARS-CoV-2 vaccination.**

|  | ***Not on JAK inhibitor*** | ***On JAK inhibitor at first vaccine dose*** | ***p-value**** |
| --- | --- | --- | --- |
| Number (n) | 16 | 24 |  |
| Age  (Years) (mean ± SD) | 66.8 ± 10.1 | 72.1 ± 9.4 | 0.10 |
| Sex (Male) | 10/16 (62.5%) | 18/24 (75%) | 0.49 |
| Myelofibrosis type |  |  | 0.10 (PMF vs secondary MF) |
| Primary MF | 13/16 (81.3%) | 13/24 (54.2%) |  |
| Post-polycythemia vera MF | 3/16 (18.8%) | 9/24 (37.5%) |  |
| Post-essential thrombocythemia MF | 0/16 (0%) | 2/24 (8.3%) |  |
| Driver mutation |  |  |  |
| JAK2 V617F | 10/16 (62.5%) | 18/24 (75%) | 0.49 |
| CALR | 4/16 (25%) | 3/24 (12.5%) | 0.41 |
| MPL | 0/16 (0%) | 2/24 (8.3%) | 0.51 |
| DIPSS+ score |  |  | **0.035** |
| Low | 2/16 (12.5%) | 3/24 (12.5%) |  |
| Intermediate-1 | 10/16 (62.5%) | 6/24 (25%) |  |
| Intermediate-2 | 4/16 (25%) | 12/24 (50%) |  |
| High | 0/16 (0%) | 3/24 (12.5%) |  |
| Median spleen size (cm below costal margin) | 3 cm (Range 0-20) | 0 cm (Range 0-15) | 0.95 |
| Median duration of JAKi treatment (years) (range) |  | 2.71 (0.01-10.9) |  |
| Mean Hb (g/L) (± SD) | 130 (±19.1) | 106 (±20.88) | **<0.001** |
| Median lymphocyte count (x10^9^/L) (IQR) | 1.55 (1.179-1.734) | 1.31 (1.009-1.872) | 0.30 |
| Median platelet count (x10^9^/L) (IQR) | 359.5 (246.6-479.2) | 134 (117.2-316.5) | **0.016** |
| Median LDH (U/L) (IQR) | 338.5 (268.6-517.4) | 574.5 (472.0-1002) | **0.011** |
| Vaccine type – First dose | AZD1222 (12)  BNT162b2 (4) | AZD1222 (20)  BNT162b2 (4) | 0.69 |
| Vaccine type – Second dose | AZD1222 (12)  BNT162b2 (4) | AZD1222 (20)  BNT162b2 (4) | 0.69 |
| Vaccine type – Third dose | BNT162b2 (12)  mRNA-1273 (4) | BNT162b2 (20)  mRNA-1273 (3) | 0.42 |

*Statistically significant differences between the two groups of patients are highlighted in bold text*.*

**Supplementary Table 3. Median values of immunological assays and statistical significance.** Interquartile ranges (IQR) given in parentheses.

|  | ***T0*** | ***T1*** | ***T2*** | ***T3*** | ***T4*** | ***p-value**** |
| --- | --- | --- | --- | --- | --- | --- |
| *αSARS-CoV-2 Spike IgG ratio (EUROIMMUN)* | | | | | | |
| JAKi (paired) | 0.090 (0.070-0.15) | 0.17 (0.11-0.32) |  |  |  | **0.017** |
|  |  | 0.17 (0.11-0.32) | 0.40 (0.22-1.69) |  |  | **<0.0001** |
|  |  |  |  | 0.29 (0.20-0.57) | 2.14 (1.27-6.40) | **0.0003** |
| Healthy control |  |  | 4.56 (4.07-8.58) |  |  | **<0.0001** (compared to JAKi patients at T2; Mann-Whitney U test) |
| Alternate therapies (paired) |  |  |  | 3.6 (0.45-4.82) | 8.55 (7.65-9.19) | **<0.0001** |
| *IFNγ SFU per 10^6^ cells (ELISpot)* | | | | | | |
| JAKi (paired) |  |  |  | 27.50 (0-42.79) | 27.50 (0-45.03) | 0.85 |
| Alternate therapies (paired) |  |  |  | 18.75 (3.75-44.96) | 30 (12.5-94.69) | 0.36 |
| *Neutralisation (IC_50_) Ancestral A.2.2* | | | | | | |
| JAKi (paired) |  |  |  | <20 (<20-<20) | <20 (<20-49.62) | **0.016** |
| Alternate therapies (paired) |  |  |  | <20 (<20-33.25) | 153.2 (83.4-601) | **0.0001** |
| JAKi vs alternate therapies at T4 |  |  |  |  |  | **0.0046** (Mann-Whitney U test) |
| *Neutralisation (IC_50_) Omicron BA.5* | | | | | | |
| JAKi (unpaired) |  |  |  | <20 (<20-<20) | <20 (<20-<20) | 0.23 (Mann-Whitney U test) |
| Alternate therapies (paired) |  |  |  | <20 (<20-<20) | 30.74 (<20-89.92) | **0.0020** |
| JAKi vs alternate therapies at T4 |  |  |  |  |  | **0.0004** (Mann-Whitney U test) |

*Statistically significant differences between the two groups of patients are highlighted in bold text.

**Supplementary Table 4.** Multiple Linear Regression predicting T3 αSARS-CoV-2 Spike IgG Ratio. The overall regression was statistically significant (R^2^=0.7044, F(7,17)=5.787, p=0.0015).

Variable β SE 95% CI *p*

Y-intercept 5.950 2.130 1.458 to 10.44 **0.0125 ***

Age (per year) -0.04899 0.03161 -0.1157 to 0.01769 0.1395

Sex [M] -1.035 0.6691 -2.447 to 0.3764 0.1402

Myelofibrosis type [SMF] 0.4636 0.6277 -0.8607 to 1.788 0.4702

DIPSS+ at V1 (per point) 0.1289 0.3182 -0.5425 to 0.8003 0.6905

Spleen at V1 (/cm) -0.03843 0.05233 -0.1488 to 0.07198 0.4728

**JAKi [on therapy] -2.735 0.7099 -4.233 to -1.237 0.0013 ****

**Lymph count (per 10^9^/L) 0.9709 0.3839 0.1609 to 1.781 0.0216 ***

*D'Agostino-Pearson omnibus (K2)=1.105 (p= 0.5755). M, male. SMF, secondary myelofibrosis.*

**Supplementary Table 5.** Multiple Linear Regression predicting T4 αSARS-CoV-2 Spike IgG Ratio. The overall regression was statistically significant (R^2^=0.4797, F(7,21)=2.766, p=0.0334).

Variable β SE 95% CI *p*

Y-intercept 12.80 3.934 4.614 to 20.98 **0.0038 ****

Age (per year) -0.02397 0.06123 -0.1513 to 0.1034 0.6994

**Sex [M] -2.633 1.189 -5.106 to -0.1598 0.0380 ***

Myelofibrosis type [SMF] -1.195 1.110 -3.504 to 1.115 0.2942

DIPSS+ at V1 (per point) -0.3782 0.5957 -1.617 to 0.8606 0.5323

Spleen at V1 (/cm) -0.1209 0.09423 -0.3169 to 0.07503 0.2133

JAKi [on therapy] -2.714 1.309 -5.436 to 0.007117 0.0505

Lymph count (per 10^9^/L) -0.08079 0.6882 -1.512 to 1.350 0.9077

*D'Agostino-Pearson omnibus (K2)=1.773 (p=0.4122)*

**Supplementary Table 6.** Multiple Linear Regression predicting T3 ELISpot IFNγ SFU per 10^6^ cells. The overall regression was not statistically significant (R^2^=0.3612, F(7,17)=0.3644, p=0.9106).

Variable β SE 95% CI *p*

Y-intercept -44.84 305.5 -689.3 to 599.6 0.8850

Age (per year) 0.6115 4.389 -8.647 to 9.870 0.8908

Sex [M] 40.83 94.64 -158.8 to 240.5 0.6716

Myelofibrosis type [SMF] 80.63 85.02 -98.76 to 260.0 0.3563

DIPSS+ at V1 (per point) 34.05 43.84 -58.44 to 126.5 0.4480

Spleen at V1 (/cm) -6.795 7.219 -22.03 to 8.436 0.3597

JAKi [on therapy] -7.231 103.6 -225.8 to 211.4 0.9452

Lymph count (per 10^9^/L) -17.01 56.72 -136.7 to 102.7 0.7679

*D'Agostino-Pearson omnibus (K2)=* *51.32 (p=* *<0.0001)*

**Supplementary Table 7.** Multiple Linear Regression model predicting T4 ELISpot IFNγ SFU per 10^6^ cells. The overall regression was not statistically significant (R^2^=0.2475, F(7,15)=0.7050, p=0.6685).

Variable β SE 95% CI *p*

Y-intercept -39.75 122.3 -300.4 to 220.9 0.7496

Age (per year) 2.610 1.830 -1.291 to 6.511 0.1744

Sex [M] -21.74 36.87 -100.3 to 56.85 0.5642

Myelofibrosis type [SMF] -6.288 34.92 -80.73 to 68.15 0.8595

DIPSS+ at V1 (per point) -27.56 16.51 -62.75 to 7.635 0.1158

Spleen at V1 (/cm) -2.178 3.472 -9.579 to 5.222 0.5398

JAKi [on therapy] 1.916 41.99 -87.58 to 91.41 0.9642

Lymph count (per 10^9^/L) -5.493 22.02 -52.44 to 41.45 0.8064

*D'Agostino-Pearson omnibus (K2)=1.673 (p=0.4333)*

**Supplementary Table 8.** Multiple Linear Regression model predicting T3 Neutralization (IC50) of Ancestral strain. The overall regression was statistically significant (R^2^=0.5410, F(7,18)=3.031, p=0.0273).

Variable β SE 95% CI *p*

Y-intercept 66.80 21.30 22.04 to 111.6 **0.0057 ****

Age (per year) -0.6037 0.3171 -1.270 to 0.06257 0.0731

Sex [M] -13.78 6.665 -27.78 to 0.2257 0.0534

Myelofibrosis type [SMF] 0.7854 6.284 -12.42 to 13.99 0.9019

DIPSS+ at V1 (per point) 3.464 3.218 -3.296 to 10.22 0.2959

Spleen at V1 (/cm) -0.4994 0.5182 -1.588 to 0.5892 0.3479

**JAKi [on therapy] -18.05 7.132 -33.04 to -3.069 0.0209 ***

Lymph count (per 10^9^/L) 0.04852 3.767 -7.867 to 7.964 0.9899

*D'Agostino-Pearson omnibus (K2)=5.342 (p=0.0692)*

**Supplementary Table 9.** Multiple Linear Regression model predicting T4 Neutralization (IC50) of Ancestral strain. The overall regression was not statistically significant (R^2^=0.2130, F(7,20)=0.7733, p=0.6164).

Variable β SE 95% CI *p*

Y-intercept 48.98 489.7 -972.4 to 1070 0.9213

Age (per year) 8.121 7.572 -7.674 to 23.92 0.2963

Sex [M] -136.4 144.6 -438.1 to 165.3 0.3569

Myelofibrosis type [SMF] -26.84 135.2 -308.8 to 255.1 0.8446

DIPSS+ at V1 (per point) -66.60 73.48 -219.9 to 86.69 0.3756

Spleen at V1 (/cm) 3.954 11.39 -19.81 to 27.72 0.7322

JAKi [on therapy] -83.64 157.9 -412.9 to 245.6 0.6021

Lymph count (per 10^9^/L) -121.0 82.77 -293.7 to 51.63 0.1592

*D'Agostino-Pearson omnibus (K2)=25.04 (p=<0.0001)*

**Supplementary Table 10.** Multiple Linear Regression model predicting T4 Neutralization (IC50) of Omicron BA.5 strain. The overall regression was statistically significant (R^2^=0.4803, , F(7,20)=2.640, p=0.0417).

Variable β SE 95% CI *p*

Y-intercept 61.42 33.59 -8.656 to 131.5 0.0825

Age (per year) 0.1163 0.5195 -0.9673 to 1.200 0.8251

Sex [M] -7.736 9.922 -28.43 to 12.96 0.4447

Myelofibrosis type [SMF] -7.353 9.273 -26.70 to 11.99 0.4371

DIPSS+ at V1 (per point) -4.657 5.041 -15.17 to 5.859 0.3666

Spleen at V1 (/cm) -0.6117 0.7815 -2.242 to 1.019 0.4430

**JAKi [on therapy] -29.20 10.83 -51.79 to -6.608 0.0139 ***

Lymph count (per 10^9^/L) -10.32 5.678 -22.17 to 1.522 0.0841

*D'Agostino-Pearson omnibus (K2)=25.04 (p=* *0.3077)*

**Supplementary Table 11.** Simple Logistic Regression model predicting vaccine response (seroconversion or effective neutralisation) by age.

Outcome β0 (95% CI) β1 (95% CI) β1 Odds ratio (95% CI) *p** Area under *p* Tjur’s R^2^ ROC

T3 αSARS-CoV-2 Spike 59.34 (0.36-22927) 0.94 (0.86-1.01) 0.94 (0.86-1.01) 0.095 0.6726 0.098 0.089

T4 αSARS-CoV-2 Spike 5.79 (-2.84-17.15) -0.047 (-0.19-0.077) 0.95 (0.82-1.08) 0.47 0.6225 0.49 0.0082

T3 Ancestral Neutralization (IC50) 4.14 (-2.17-11.35) -0.084 (-0.20-0.0086) 0.92 (0.82-1.01) 0.076 0.6759 0.18 0.12

T4 Ancestral Neutralization (IC50) 3.53 (-2.02-9.99) -0.034 (-0.123-0.045) 0.97 (0.88-1.05) 0.40 0.6008 0.37 0.016

T4 BA.5 Neutralization (IC50) 2.33 (-2.60-7.72) -0.040 (-0.11-0.030) 0.96 (0.89-1.03) 0.27 0.5942 0.35 0.035

**Likelihood ratio test.*

**Supplementary Table 12.** Simple Logistic Regression model predicting vaccine response (seroconversion or effective neutralisation) by sex. Empty rows did not converge.

Outcome β0 (95% CI) β1 (95% CI) β1 Odds ratio (95% CI) *p** Area under *p* Tjur’s R^2^ ROC

T3 αSARS-CoV-2 Spike 0.41 (-0.85-1.77) -0.96 (-2.58-0.55) 0.38 (0.076-1.73) 0.21 0.6032 0.32 0.04877

T4 αSARS-CoV-2 Spike

T3 Ancestral Neutralization (IC50) -0.85 (-2.38-0.43) -1.05 (-2.94-0.82) 0.35 (0.053-2.27) 0.26 0.6204 0.36 0.04082

T4 Ancestral Neutralization (IC50) 1.50 (0.15-3.39) -0.56 (-2.59 to 1.09) 0.57 (0.075-2.98) 0.52 0.5556 0.62 0.01091

T4 BA.5 Neutralization (IC50) 0.18 (-1.02-1.43) -0.94 (-2.44-0.51) 0.39 (0.087-1.67) 0.20 0.6006 0.31 0.04538

**Likelihood ratio test.*

**Supplementary Table 13.** Simple Logistic Regression model predicting vaccine response (seroconversion or effective neutralisation) by DIPSS+ score.

Outcome β0 (95% CI) β1 (95% CI) β1 Odds ratio (95% CI) *p** Area under *p* Tjur’s R^2^ ROC

T3 αSARS-CoV-2 Spike 0.63 (-0.70-2.09) -0.57 (-1.46-0.15) 0.56 (0.23-1.16) 0.12 0.6468 0.16 0.07009

T4 αSARS-CoV-2 Spike 2.54 (0.69-5.17) -0.068 (-1.14-1.14) 0.94 (0.32-3.13) 0.90 0.56 0.74 0.0002470

T3 Ancestral Neutralization (IC50) -0.97 (-2.7-0.61) -0.36 (-1.48-0.54) 0.70 (0.23-1.71) 0.45 0.59 0.50 0.01703

T4 Ancestral Neutralization (IC50) 2.37 (0.88-4.31) -0.70(-1.54-0.0047) 0.49 (0.21-1.00) 0.052 0.7181 0.053 0.09860

T4 BA.5 Neutralization (IC50) 0.48 (-0.74-1.79) -0.61 (-1.41-0.052) 0.54 (0.24-1.053) 0.072 0.6672 0.095 0.08559

**Likelihood ratio test.*

**Supplementary Table 14.** Simple Logistic Regression model predicting vaccine response (seroconversion or effective neutralisation) by lymphocyte count.

Outcome β0 (95% CI) β1 (95% CI) β1 Odds ratio (95% CI) *p** Area under *p* Tjur’s R^2^ ROC

T3 αSARS-CoV-2 Spike -1.82 (-4.14-0.015) 0.60 (-0.074-2.34) 2.65 (0.93-10.38) 0.070 0.6726 0.098 0.09438

T4 αSARS-CoV-2 Spike 2.37 (-0.35-5.34) 0.038 (-1.44-2.10) 1.04 (0.24-8.20) 0.96 0.5392 0.82 2.926e-005

T3 Ancestral Neutralization (IC50) -1.48 (-3.68-0.54) -0.016 (-1.34-1.13) 0.984 (0.26-3.11) 0.98 0.5802 0.54 1.625e-005

T4 Ancestral Neutralization (IC50) 1.28 (-0.45-3.12) -0.12 ( -1.15-1.01) 0.88 (0.32-2.74) 0.82 0.5576 0.61 0.001307

T4 BA.5 Neutralization (IC50) 0.68 (-0.97-2.57) -0.79 (-2.11-0.25) 0.45 (0.12-1.28) 0.14 0.5909 0.36 0.05322

**Likelihood ratio test.*

**Supplementary Table 15.** Simple Logistic Regression model predicting vaccine response (seroconversion or effective neutralisation) by spleen size [cm].

Outcome β0 (95% CI) β1 (95% CI) β1 Odds ratio (95% CI) *p** Area under *p* Tjur’s R^2^ ROC

T3 αSARS-CoV-2 Spike -0.26 (-1.28 to 0.73) 0.0041 (-0.14 to 0.15) 1.00 (0.87 to 1.16) 0.95 0.5260 0.83 0.07009

T4 αSARS-CoV-2 Spike 2.23 (0.91-4.12) -0.015 (-0.21-0.25) 0.99 (0.81-1.28) 0.89 0.53 0.89 0.0007906

T3 Ancestral Neutralization (IC50) -1.16 (-2.55 to -0.025) -0.20 (-0.77-0.058) 0.82 (0.46-1.06) 0.16 0.69 0.23 0.05987

T4 Ancestral Neutralization (IC50) 0.96 (-0.018-2.08) -0.010 (-1.54-0.15) 0.99 (0.86-1.16) 0.89 0.5250 0.84 0.0006433

T4 BA.5 Neutralization (IC50) -0.65 (-1.7-0.31) -0.072 (-0.28-0.082) 0.93 (0.76-1.09) 0.38 0.5938 0.45 0.02401

**Likelihood ratio test.*

**Supplementary Table 16.** Simple Logistic Regression model predicting vaccine response (seroconversion or effective neutralisation) by presence of JAKi therapy. Empty rows were not converged.

Outcome β0 (95% CI) β1 (95% CI) β1 Odds ratio (95% CI) *p** Area under *p* Tjur’s R^2^ ROC

**T3 αSARS-CoV-2 Spike** 0.69 (-0.34-1.86) -1.87 (-3.54 to -0.39) **0.15 (0.029-0.68)**  **0.013**  0.7183 **0.0367** 0.1883

T4 αSARS-CoV-2 Spike

T3 Ancestral Neutralization (IC50)

**T4 Ancestral Neutralization (IC50)** 2.70 (1.11-5.60) -2.30 (-5.29 to -0.42) **0.10 (0.0050-0.65)** **0.014** 0.7222 **0.049** 0.1500

**T4 BA.5 Neutralization (IC50)** 0.79 (-0.22-1.94) -2.52 (-4.31 to -1.01) **0.080 (0.013-0.37)** **0.0008** 0.7792 **0.0053** 0.3002

**Likelihood ratio test.*

**Supplementary Table 17.** Characteristics of responders vs non-responders by αSARS-CoV-2 Spike (IgG ratio ≥1.1) at T3 and T4.

|  | ***T3*** | | | |  | ***T4*** | | | |
| --- | --- | --- | --- | --- | --- | --- | --- | --- | --- |
|  | ***Non-responders*** | ***Responders*** | ***Relative Risk^*** | ***p-value**** |  | ***Non-responders*** | ***Responders*** | ***Relative Risk^*** | ***p-value**** |
| *n* | *18* | *14* |  |  |  | *3* | *34* |  |  |
| Median age (Years) | 73 | 66 |  | 0.10 |  | 75 | 69.5 |  | 0.51 |
| Sex (Male/Female) | 14/4 | 8/6 | 1.59 (0.80-3.95) | 0.27 |  | 3 | 23 | - | 0.54 |
| Myelofibrosis type [SMF] | 6 | 5 | 0.95 (0.46-1.75) | >0.99 |  | 2 | 11 | 3.69 (0.52-26.29) | 0.28 |
| **On JAKi therapy** | **13** | **4** | **2.29 (1.17-5.20)** | **0.031** |  | 3 | 18 | - | 0.24 |
| Median spleen size (cm below costal margin) | 2.5 cm (0-8) | 0 cm (0-6) |  | 0.83 |  | 0 (0-14)** | 0.5 (0-8) |  | 0.91 |
| Median lymphocyte count (x10^9^/L) (IQR) | 1.31 (0.96-1.92) | 1.61 (1.50-2.25) |  | 0.10 |  | 1.55 (1.30-1.58)** | 1.47 (1.01-1.83) |  | 0.85 |

*Statistically significant differences between the two groups of patients are highlighted in bold text. ^Relative risk for non-response. Values in parentheses reflect interquartile ranges or confidence intervals, where appropriate. Dash indicates values that include infinity. **These values in parentheses represent range.

**Supplementary Table 18.** Characteristics of responders vs non-responders by neutralising antibodies against Ancestral strain (see *Methods* above) at T3 and T4.

|  | ***T3*** | |  | | |  | ***T4*** |  | | |
| --- | --- | --- | --- | --- | --- | --- | --- | --- | --- | --- |
|  | ***Non-responders*** | ***Responders*** | | ***Relative Risk^*** | ***p-value**** |  | ***Non-responders*** | ***Responders*** | ***Relative Risk^*** | ***p-value**** |
| *n* | *27* | *6* | |  |  |  | *9* | *27* |  |  |
| Median age (Years) | 70 (65-78) | 62 (51-76.75) | |  | 0.19 |  | 71 (67.5-77) | 69 (61-77) |  | 0.38 |
| Sex (Male/Female) | 20/7 | 3/3 | | 1.24 (0.87-2.22) | 0.34 |  | 7/2 | 18/9 | 1.54 (0.46-6.07) | 0.69 |
| Myelofibrosis type [SMF] | 10 | 1 | | 1.18 (0.78-1.65) | 0.64 |  | 4 | 8 | 1.60 (0.53-4.57) | 0.44 |
| **On JAKi therapy** | **18** | **0** | | **1.67 (1.67-1.92)** | **0.0045** |  | **8** | **12** | **6.40 (1.25-37.0)** | **0.026** |
| Median spleen size (cm below costal margin) | 3 (0-8.25) | 0 cm (0-3.75) | |  | 0.24 |  | 2.5 (0-8) | 0 (0-7.5) |  | 0.85 |
| Median lymphocyte count (x10^9^/L) (IQR) | 1.53 (1.10-1.99) | 1.63 (1.29-1.91) | |  | 0.56 |  | 1.55 (1.15-2.11) | 1.42 (1.02-1.64) |  | 0.62 |

*Statistically significant differences between the two groups of patients are highlighted in bold text. ^Relative risk for non-response. Values in parentheses reflect interquartile ranges or confidence intervals, where appropriate.

**Supplementary Table 19.** Characteristics of responders vs non-responders by neutralising antibodies against Omicron BA.5 strain (see *Methods* above) at T4 (no patient had neutralising antibodies at T3).

|  | ***T3*** | |  | | |  | ***T4*** |  | | |
| --- | --- | --- | --- | --- | --- | --- | --- | --- | --- | --- |
|  | ***Non-responders*** | ***Responders*** | | ***Relative Risk^*** | ***p-value**** |  | ***Non-responders*** | ***Responders*** | ***Relative Risk^*** | ***p-value**** |
| *n* |  |  | |  |  |  | *22* | *14* |  |  |
| Median age (Years) |  |  | |  |  |  | 70.5 (64.25-78.25) | 67 (60.5-75.25) |  | 0.35 |
| Sex (Male/Female) |  |  | |  |  |  | 17/5 | 8/6 | 1.50 (0.83-3.31) | 0.27 |
| Myelofibrosis type [SMF] |  |  | |  |  |  | 9 | 3 | 1.39 (0.79-2.28) | 0.29 |
| **On JAKi therapy** |  |  | |  |  |  | **17** | **3** | **2.72 (1.44-6.10)** | **0.0017** |
| Median spleen size (cm below costal margin) |  |  | |  |  |  | 0.5 (0-8) | 0 (0-5) |  | 0.43 |
| Median lymphocyte count (x10^9^/L) (IQR) |  |  | |  |  |  | 1.47 (1.14-2.05) | 1.32 (0.72-1.68) |  | 0.37 |

*Statistically significant differences between the two groups of patients are highlighted in bold text. ^Relative risk for non-response. Values in parentheses reflect interquartile ranges or confidence intervals, where appropriate. ^#^ Exploratory variables analysed against an α significance level of 0.01. Not all patients had biochemistry measurements.

**Supplementary Table 20.** Median (and IQR) serological values for patients who later developed COVID-19 clinical infection compared to those who did not (exploratory analysis).

**Measure COVID-19 infection (Median, IQR) (*n*=10) Not infected (Median, IQR) (*n*=22)**  ***p-value***

T3 αSARS-CoV-2 Spike 0.42 (0.24 to 2.64) 0.73 (0.29 to 3.92) 0.49

T4 αSARS-CoV-2 Spike 5.83 (1.41 to 7.93) 7.83 (3.49 to 8.78) 0.18

T3 Ancestral Neutralization (IC50) 0 (0 to 0) 0 (0 to 20.30) 0.14

T4 Ancestral Neutralization (IC50) 38.0 (0 to 145.8) 117.3 (27.5 to 277.2) 0.15

T4 BA.5 Neutralization (IC50) 0 (0 to 6.9) 0 (0 to 53.9) 0.17

Supplementary Figures

**Supplementary Figure 1. Serological response to SARS-CoV-2 Spike protein following COVID-19 vaccination is impaired by JAKi in MF patients but is partially overcome by a third vaccine dose. (A)** MF patients on JAKi showed significantly reduced αSARS-CoV-2 Spike IgG ratio compared to MF patients not on JAKi (median 0.29 vs 3.6, p=0.0026) and most JAKi patients did not seroconvert (values below the dashed line). **(B)** A significant difference between the two groups was maintained following dose three (4.0 vs 8.6, p<0.0001) but this difference was less pronounced and the majority of JAKi patients became seropositive (above the dashed line). **(C)** JAKi patients showed poor median response to vaccination at T1 (0.17), T2 (0.40), and T3 (0.29). However, a third vaccine dose was substantially more effective than previous doses in eliciting a response, raising the level at T4 to 4.0 (from left to right, top bars represent p=0.0023 and 0.040, and bottom bars p=0.74, 0.61, >0.99, and 0.0088, respectively) (Kruskal-Wallis test with Dunn’s correction). **(D)** Seroconversion (anti-Spike IgG ratio ≥1.1) was substantially improved after third vaccine dose (24% vs 82%, p=0.0002; not indicated on chart) such that difference between JAKi and non-JAKi seroconversion at T4 was not significant. At T3 (before third vaccine dose) substantially fewer JAKi patients seroconverted (24% vs 67%, p=0.031). **(E)** Median αSARS-CoV-2 Spike IgG ratio at T3 was lower in JAKi patients (0.74) compared to MF patients on hydroxyurea (HU) (2.60, p=0.067) and MF patients on neither treatment (3.5, p=0.044) (Dunn’s multiple comparison test). **(F)** Median αSARS-CoV-2 Spike IgG ratio at T4 was lower in JAKi patients (4.0) compared to MF patients on hydroxyurea (HU) (8.7, p=0.012) and MF patients on neither treatment (8.5, p=0.0055) (Dunn’s multiple comparison test). No difference was observed between HU and no therapy patients (p>0.99). Horizontal bars represent median, and error bars represent interquartile range**.** Dashed lines represent seroconversion threshold. **ns, non-significant; *p<0.05; **p<0.01; ***p<0.001; ****p<0.0001.**

**Supplementary Figure 2. Serological neutralization of live SARS-CoV-2 virus (Ancestral and Omicron BA.5) in MF patients stratified by JAKi therapy, HU therapy or supportive care/no treatment. (A)** At T3, JAKi patients showed a median IC50 neutralization against Ancestral strain of <20 (IQR <20 to <20), compared to patients on HU (<20; IQR <20 to <20; p>0.99) and no therapy (23.18, IQR <20 to 37.32; p=0.0007). **(B)** At T4, JAKi patients showed a lower median IC50 neutralisation against Ancestral strain of 43.24 (IQR <20 to <103.9), compared to patients on HU (158.2; IQR 42.98-534.5; p=0.20) and no therapy (168.7, IQR 125.2-1074; p=0.024) **(C)** At T4, JAKi patients showed a smaller median IC50 neutralisation against Omicron BA.5 strain of <20 (IQR <20 to <20), compared to patients on HU (35.91, IQR <20 to 88.09; p=0.11) and no therapy (41.22, IQR 28.24-180.9; p=0.0018). Significant difference between groups by Kruskal-Wallis test with Dunn’s correction for multiple comparisons. Horizontal bars represent median, and error bars represent interquartile range**. ns, non-significant; *p<0.05; **p<0.01; ***p<0.001.**

**Supplementary Figure 3. Patients who were not on JAKi therapy at time of first vaccine dose, but later went on JAKi by the time of second (n=1) or third (n=3) vaccine dose, showed better serological response compared to patients who were already on JAKi at time of first dose. (A)** Median anti-S IgG ratio for such patients was 4.105 (IQR 1.38-7.62) at T3, compared to 0.29 (IQR 0.22-0.85) for MF patients already on JAKi at first vaccine dose (p=0.0058). **(B)** MF patients on alternate therapy who never went on JAKi during study had median response 3.60 (IQR 0.33-4.59). **(C-D)** MF patients who later started JAKi still had higher median response at T4, but this was no longer statistically significant, likely due to small numbers and improvement in response in JAKi patients. Horizontal bars represent median, and error bars represent interquartile range**.** Dashed lines represent seroconversion threshold. **ns, non-significant; *p<0.05; **p<0.01; ***p<0.001; ****p<0.0001.**

**Supplementary Figure 4. Measured immune serology in patients who later developed COVID-19 infection compared to those without clinical infection (exploratory analysis). (A)** Anti-Spike IgG at T3 and **(B)** T4 (after third vaccine dose). **(C)** IC50 Neutralisation against Ancestral A.2.2 at T3 and **(D)** T4. **(E)** IC50 Neutralisation against Omicron BA.5 strain. P values for the null hypothesis (no difference between groups) is indicated. The study was not powered to investigate serological differences between those who developed clinical infection and those who did not. Horizontal bars represent median, and error bars represent interquartile range**.**

Data Sharing and Supplementary References

**Data Sharing Statement**

For original data, please contact A. Al.

**Supplementary References**

1. Beavis KG, Matushek SM, Abeleda APF, Bethel C, Hunt C, Gillen S, et al. Evaluation of the EUROIMMUN Anti-SARS-CoV-2 ELISA Assay for detection of IgA and IgG antibodies. J Clin Virol. 2020 Aug;129:104468.

2. Aggarwal A, Stella AO, Walker G, Akerman A, Esneau C, Milogiannakis V, et al. Platform for isolation and characterization of SARS-CoV-2 variants enables rapid characterization of Omicron in Australia. Nat Microbiol. 2022 Jun;7(6):896–908.

3. Aggarwal A, Akerman A, Milogiannakis V, Silva MR, Walker G, Stella AO, et al. SARS-CoV-2 Omicron BA.5: Evolving tropism and evasion of potent humoral responses and resistance to clinical immunotherapeutics relative to viral variants of concern. EBioMedicine. 2022 Oct;84:104270.

4. Tea F, Ospina Stella A, Aggarwal A, Ross Darley D, Pilli D, Vitale D, et al. SARS-CoV-2 neutralizing antibodies: Longevity, breadth, and evasion by emerging viral variants. PLoS Med. 2021 Jul;18(7):e1003656.

5. Khoury DS, Cromer D, Reynaldi A, Schlub TE, Wheatley AK, Juno JA, et al. Neutralizing antibody levels are highly predictive of immune protection from symptomatic SARS-CoV-2 infection. Nat Med. 2021 Jul;27(7):1205–11.

6. Mattiuzzo G, Bentley EM, Hassall M, Routley S, Richardson S, Bernasconi V, et al. WHO/BS.2020.2403 Establishment of the WHO International Standard and Reference Panel for anti-SARS-CoV-2 antibody [Internet]. WHO, Geneva; 2020 [cited 2023 Feb 6]. Available from: https://www.who.int/publications/m/item/WHO-BS-2020.2403

7. Perkins GB, Tunbridge M, Salehi T, Chai CS, Kireta S, Johnston J, et al. Concurrent vaccination of kidney transplant recipients and close household cohabitants against COVID-19. Kidney Int. 2022 May;101(5):1077–80.
